# Genome-wide allele and haplotype-sharing patterns suggested one unique Hmong–Mein-related lineage and biological adaptation history in Southwest China

**DOI:** 10.1186/s40246-023-00452-0

**Published:** 2023-01-31

**Authors:** Jiawen Wang, Lin Yang, Shuhan Duan, Qiuxia Sun, Youjing Li, Jun Wu, Wenxin Wu, Zheng Wang, Yan Liu, Renkuan Tang, Junbao Yang, Chao Liu, Buhong Yuan, Daoyong Wang, Jianwei Xu, Mengge Wang, Guanglin He

**Affiliations:** 1 Institute of Rare Diseases, West China Hospital of Sichuan University, Sichuan University, Chengdu, 610041 China; 2 College of Forensic Medicine, Guizhou Medical University, Guiyang, 550004 China; 3 School of Basic Medical Sciences, North Sichuan Medical College, Nanchong, 637000 China; 4 Department of Forensic Medicine, College of Basic Medicine, Chongqing Medical University, Chongqing, 400331 China; 5 Congjiang People’s Hospital, Congjiang, 557499 China; 6 Department of Pharmacology, School of Basic Medicine, Guizhou Medical University, Guiyang, 550004 China; 7 Faculty of Forensic Medicine, Zhongshan School of Medicine, Sun Yat-Sen University, Guangzhou, 510275 China; 8 Institute of Forensic Medicine, West China School of Basic Medical Sciences and Forensic Medicine, Sichuan University, Chengdu, 610000 China; 9 Longli People’s Hospital, Longli, 551299 China; 10 Nayong Guohua Yixin Hospital, Nayong, 553306 China

**Keywords:** Genetic structure, Evolutionary history, Miao, Admixture model, Biological adaptation

## Abstract

**Background:**

Fine-scale genetic structure of ethnolinguistically diverse Chinese populations can fill the gap in the missing diversity and evolutionary landscape of East Asians, particularly for anthropologically informed Chinese minorities. Hmong–Mien (HM) people were one of the most significant indigenous populations in South China and Southeast Asia, which were suggested to be the descendants of the ancient Yangtze rice farmers based on linguistic and archeological evidence. However, their deep population history and biological adaptative features remained to be fully characterized.

**Objectives:**

To explore the evolutionary and adaptive characteristics of the Miao people, we genotyped genome-wide SNP data in Guizhou HM-speaking populations and merged it with modern and ancient reference populations via a comprehensive population genetic analysis and evolutionary admixture modeling.

**Results:**

The overall genetic admixture landscape of Guizhou Miao showed genetic differentiation between them and other linguistically diverse Guizhou populations. Admixture models further confirmed that Miao people derived their primary ancestry from geographically close Guangxi Gaohuahua people. The estimated identity by descent and effective population size confirmed a plausible population bottleneck, contributing to their unique genetic diversity and population structure patterns. We finally identified several natural selection candidate genes associated with several biological pathways.

**Conclusions:**

Guizhou Miao possessed a specific genetic structure and harbored a close genetic relationship with geographically close southern Chinese indigenous populations and Guangxi historical people. Miao people derived their major ancestry from geographically close Guangxi Gaohuahua people and experienced a plausible population bottleneck which contributed to the unique pattern of their genetic diversity and structure. Future ancient DNA from Shijiahe and Qujialing will provide new insights into the origin of the Miao people.

**Supplementary Information:**

The online version contains supplementary material available at 10.1186/s40246-023-00452-0.

## Introduction

The complex human admixture history in Eastern Eurasia has resulted in the formation of Mongolic-, Turkic-, and Tungusic-speaking populations in present-day northern East Asia, Sino-Tibetan (ST)-speaking populations across East Asia, Hmong–Mien (HM), Tai–Kadai (TK), Austronesian (AN), and Austroasiatic (AA)-speaking populations in southern East Asia and Southeast Asia. Several comprehensive studies have been conducted to characterize East Asia’s linguistic and genetic history [1–7]. These studies found that geographic isolation, population expansion, and cultural interaction have shaped the genetic and linguistic landscape of modern and ancient Chinese populations [5, 8, 9]. Besides, agriculture innovation from the Yellow River Basin and Yangtze River Basin significantly influenced the population formation of ST and southern Chinese indigenous TK, HM, AA, and AN people [10]. The ancestor of modern HM people and their cultures (beliefs, languages, and technologies) are suggested to be associated with the origin of rice farming from the middle Yangtze River Basin. Modern HM people are predominantly living in mountainous areas (Guizhou, Hunan, Hubei, Sichuan, Yunnan, Guangxi, and Hainan) in southern East Asia [11–13] and northern Mainland Southeast Asia [4]. Recent archeological, genetic, and historical evidence has further revealed that HM groups might originate from the Neolithic Daxi groups (~ 5000 to 3300 BCE) in the middle reaches of the Yangtze River [14–17] and migrated westward and southward into the Yungui Plateau due to climate changes and warfare. Historic documents suggested that the migrations and admixture of ancient source populations facilitated the formation of the present-day distributions of HM groups in Southeast Asia (mainly in Laos and Vietnam), Europe, the USA, and Australia during the Ming and Qing dynasties [18–21].

Southwest China possessed rich archaeological cultures, including the Shijiahe, Qujialing, Daxi cultures and others. Much evidence has linked the origin of HM people and the ancient ancestors of Yangtze Rice farmers. Li et al. conducted a Y-chromosome-based ancient DNA study to explore the genetic relationship between HM-speaking (Miao, She, and Yao) and geographically close modern and ancient East Asians [14]. Their inferred population evolutionary model based on the Y-chromosome haplogroup O2a2a1a2-M7 suggested that the 5000-year-old Daxi people may have shared a common ancestor with the contemporary Miao people [14], which provided a critical piece of genetic evidence of common origin between ancient Daxi people and modern Miao people. HM-speaking populations currently have their specific language (Hmong and Mien) and culture. Still, few written records of the Hmong language were conserved, which was spread mainly relying on myths and legends and oral transmission. Here, we aimed to present a deeper and more comprehensive evolutionary history of the HM-speaking populations. A fine-scale genetic study focused on genetic origins is beneficial to explore the cultural roots of the HM-speaking people and their admixed history and to gain a comprehensive understanding of the migration events and admixture times in East and Southeast Asian history.

Research on the mystery of HM people and their language family has increased with advances in computational biology and molecular biology techniques. Previous studies have primarily focused on forensic-related autosomal short tandem repeats (STRs), insertion/deletions (Indels), Y-chromosomal, and mitochondrial single nucleotide polymorphisms (SNPs) markers [13, 22–26]. These studies have been dwarfed in studying the fine-scale genetic structure and exploring the history of the genetic mixing process. Recently, the reported 500-year-old Gaohuahua population was found to be genetically related to the HM ancestry [8]. Still, a recent study of genome-wide SNPs in the present-day HM populations showed that the Libo Yao is more representative of the ancestral lineage of HM groups than the 500-year-old Gaohuahua [27]. There is a growing number of genome-wide studies of HM-speaking populations, and they all revealed a north–south mixing pattern of HM-speaking people and the existence of genetic substructures among HM-speaking populations from different regions [27–29]. Gao et al. extrapolated the divergence time of HM-speaking  people and found that the separation of Yao from other HM groups was earlier than that between Miao and She. These findings also supported that the Yao was closer related to populations belonging to the TK language family than the Miao and She [30]. Liu et al. collected genome-wide SNP data from three representative Miao groups in Sichuan Province and merged them with data from 144 Miaos from 13 regions to comprehensively characterize the biological adaptations of the Miao people during the migration [31]. The findings revealed high genetic similarity among HM populations from different regions in southwestern China and Southeast Asia, but subtle genetic differences between each other.

Although there are many studies on the genetic origins, admixture history and biological adaptations of Miao populations, limited genetic studies focused on the fine-scale genetic structure and admixture processes of geographically diverse Guizhou Miao people have been performed, especially complex admixture modeling based on the haplotype information. Guizhou, located on the Yungui Plateau with a high topography in the west and a low topography in the east, is known to be one of the provinces rich in ethnic diversity. Its complex topography has laid the foundation for its ethnic diversity. For example, the Miao people in Congjiang/Nayong/Longli/Duyun County from Guizhou Province are excellent sites for studying and supplementing genetic data of HM-speaking populations. Many villages still preserve the traditional Hmong language and culture, which have not been reported in detail. We collected genetic data from the Guizhou Miao people and conducted deep genomic research, which allowed Miao and other ethnic minorities to benefit from participating in precision medicine and achieve clinical precision treatment [32]. Generally, we collected 67 Guizhou Miao samples and merged the newly generated data with data from HM populations from the HGDP to analyze the genetic structure and admixture history of the HM-speaking populations and explore biological adaptations. We also estimated the identity by descent (IBD) and calculated the effective population size to explore the genetic differences between the Miao and other ethnic groups.

## Results

### The general structure of HM populations in the context of East and Southeast Asian populations

**Fig. 1:**
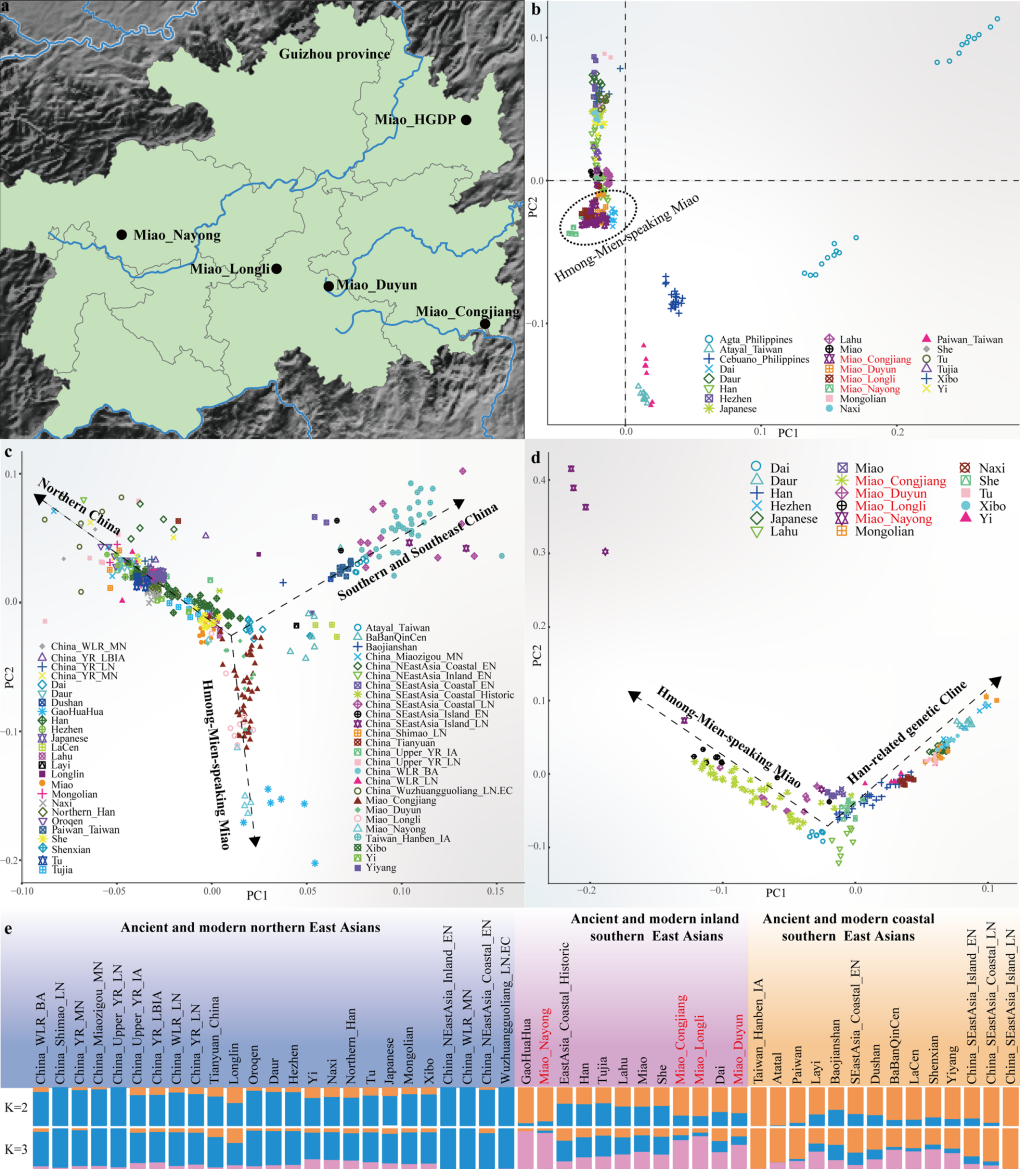
The genetic affinity between HM populations and ancient and modern East Asians. **a** The geographical positions of our studied HM populations and reference populations; **b** principal component analysis (PCA) of 22 populations at the East and Southeast Asian scale; **c** PCA analysis of 51 ancient and modern populations at the East Asian scale; and **d** PCA analysis of 17 modern populations at the East Asian scale; **e** ADMIXTURE results showed genetic similarity between ancient and modern populations at the East Asian scale with our studied HM people in the *K* value = 2 and 3

We genotyped approximately 669,896 genome-wide SNPs in 67 Miao individuals from four populations  [Miao_Congjiang (CJM), Miao_Nayong (NYM), Miao_Duyun (DYM), and Miao_Longli (LLM)] in Guizhou Province (Fig. 1a). We then merged our data with published modern and ancient worldwide populations from the Whole-Genome Sequencing (WGS), Human Origins (HO), and 1240K datasets. These datasets included African, North American, South Asian, Southeast Asian, European, Central Asian, and Oceanian populations, as well as ancient populations from Northwest China, Northeast China, Middle Yellow River, Southeast China, and the ancient Gaohuahua, BaBanQinCen, and Longlin people in Guangxi; modern Altaic, ST, and HM-speaking populations. We performed a PCA analysis to explore the general relationship between the HM-speaking populations and East and Southeast Asian populations. We observed that the population clustering patterns were consistent with the geographic and linguistic distribution. In the East and Southeast Asian PCA (Fig. 1b), we observed that the four Miao populations clustered together to form a unique cluster and were closely related to the Miao and She from the HGDP project, followed by the Han, Tu, Dai, and Lahu. And compared with NYM, the other three HM-speaking populations (DYM, LLM, and CJM) gathered more closely, and they were closely related to Dai, indicating that DYM, LLM, CJM, and Dai have more genetic exchanges in the process of population evolution and cultural exchange.

**Fig. 2:**
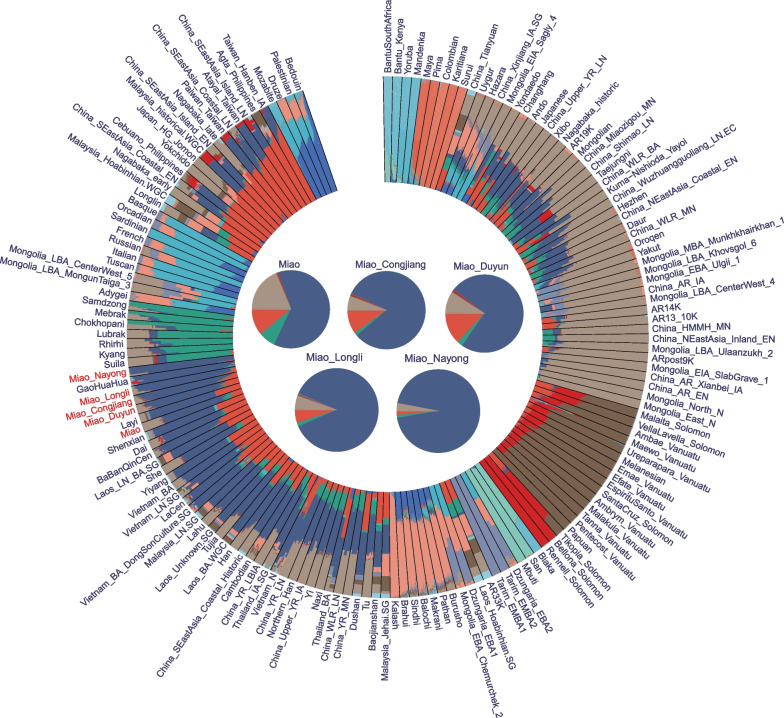
The general structure of East and Southeast Asian populations. We merged our data with published modern and ancient global populations from the WGS and 1240K datasets. We conducted an ADMIXTURE analysis based on the combined dataset of 121 ancient and modern worldwide populations (*K* = 13)

To identify the genetic affinity of our studied Miao people with other geographically and linguistically distributed worldwide populations, we conducted an ADMIXTURE analysis based on the combined dataset of 121 ancient and modern worldwide populations. When *K* = 13, five ancestral components (dark blue, orange, light gray, light green, and red) were found in HM populations (Fig. 2). The dark blue component reached the largest proportion in the NYM and Gaohuahua in southern East Asia. The orange component reached the largest proportion in the Taiwan_Hanben_IA in southern China and the Agta_Philippines in Southeast Asia. The light gray component reached the largest proportion in the Mongolia_North_N and Mongolia_East_N, the light green component reached its maximum proportion in Nepal’s Samdzong and Mebrak populations, and the red component reached its maximum proportion in the Bellona_Solomon and Rennell_Solomon populations of Oceania. We also found that the HM populations (She and Miao) from the HGDP contained a higher proportion of ancestral components related to northern East Asian compared with the four Miao populations we studied, indicating that they were significantly influenced by incoming northern East Asian gene flow. Interestingly, we also found genetic differences among our studied Miao populations. The dark blue ancestral component in NYM, LLM, CJM, and DYM were in descending order, indicating that NYM is more representative of the ancestors of the HM-speaking populations than the other three Miao groups and even more representative of the ancestors of the HM-speaking people than Gaohuahua, as previously commonly believed.

### The genetic affinity between HM populations and ancient East Asians

To visualize the genetic relationship between HM populations and ancient East Asians, we further performed PCA analysis on 51 populations from ancient and modern East Asia based on the merged 1240K dataset (Fig. 1c). We observed that our studied Miaos formed a unique genetic branch that clustered closely with HM populations from the HGDP and Dai and ST-speaking populations compared to ancient and modern people from northern China and ancient coastal and TK populations from southeastern China. The HM populations were more related to Gaohuahua from Guangxi 500 years ago and BaBanQinCen from Guangxi 1500 years ago than all other ancient East Asians. And we found that NYM was clustered closely with the Gaohuahua compared with DYM, LLM, and CJM.

We conducted a model-based ADMIXTURE clustering analysis to profile the ancestral components and genetic resemblance of our studied Miao with geographically close ancient and present-day populations. At *K* = 2 (Fig. 1e), we observed the minimum CV value. We observed two ancestral components, with the blue component reaching a maximum in Neolithic ancients from the middle reaches of the Yellow River and northwestern ancients, and the orange component reaching its maximum in populations from the southern China, especially in the ancient southeastern coastal people and Hanben_Taiwan populations. We found that the proportion of these two ancestral components in the HM populations from the HGDP was almost equal to that in Han, Tujia, and Lahu, which were genetically related to the coastal people and Han populations in southern China, indicating that the HM populations from the HGDP were heavily Sinicized. We also found that all Miaos in our study showed fewer blue ancestral components and more orange ancestral components, especially NYM, which had almost all orange ancestral components, indicating that NYM was less influenced by northern gene flow.

When *K* = 3, three ancestral components of blue, orange, and pink were observed in the HM populations (Fig. 1e). The blue component reached its maximum in the ancients from Neolithic Northern Inland regions, Western Liao River, and Shaanxi Province in the middle reaches of the Yellow River. And the orange component reached its maximum in the southern Chinese islanders and the Hanben_Taiwan, and the emerging pink component reached its maximum in the Gaohuahua. Compared to all reference populations, our studied Miaos had more pink ancestral components, suggesting that the Gaohuahua population was likely to be the ancestor proxy of the HM populations. Compared with HM populations from HGDP, our studied Miaos had less gene flow from the ancient genetic lineage from Northern East Asia, especially NYM. NYM is more representative of the ancestral source of HM populations than DYM, LLM, and CJM.

### Genetic affinity of HM populations with southern East Asians and Southeast Asian populations

The PCA analysis in the context of East Asians revealed that HM-speaking populations formed a separate genetic branch (Fig. 1d). In general, our studied Miaos clustered closely with the HM populations (Miao and She), Dai, and Lahu from HGDP. We also found that the HM populations from the HGDP largely overlapped with Han-associated populations, indicating that Han-associated genetic materials largely influenced the HM populations from the HGDP compared to our studied Miaos. Interestingly, NYM was distant from the other three Miao populations and Han-related genetic lineages. DYM and CJM were located at the intersection of HM and Han-related genetic lineages, suggesting that Han populations influenced these two studied Miao populations significantly.

**Fig. 3:**
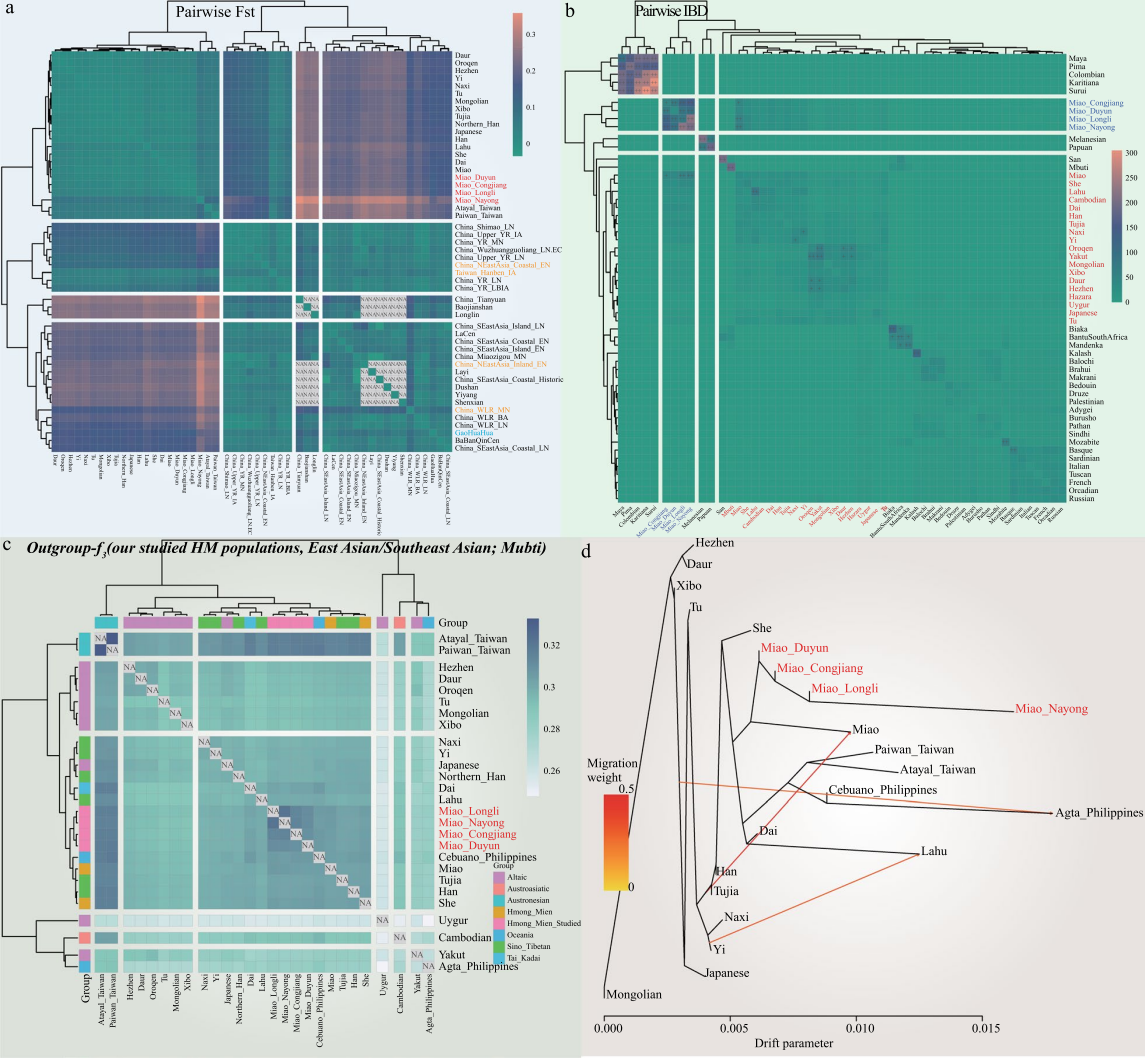
Genetic affinity of HM populations with East and Southeast Asian populations. **a**, Heatmap of the pairwise Fst genetic distances among 51 worldwide populations. NA: Not applicable due to the small sample size of the ancient population. **b** Shared allele counts, including worldwide representative 54 populations. +  + means a strong positive result with the IBD number over 50. **c** Shared genetic drift inferred from out-group *f*_3_-statistics in the form *f*_3_(studied Miao, East Asian/Southeast Asian; Mbuti), including 27 populations in East Asia/Southeast Asia. **d** TreeMix-based maximum likelihood tree with three migration events revealed the genetic relationship between East Asians/Southeast Asians

We carried out admixture-*f*_3_ in the form *f*_3_(X, Y; studied Miao) to test mixed signals using all possible ancestral source pairs. LLM produced significant negative *Z*-values (*Z* < −3) when combined with the northern sources related to ST (Han, Tujia, Naxi, and Yi), Altaic (Daur, Mongolian, Hezhen, and Xibo), and southern sources related to Guizhou aborigines (Guizhou Han, Chuanqing, etc.) and Southeast Asian populations (Additional file 1: Table S1). When DYM was used as the targeted population, NYM, LLM, and CJM as one source and combined with other East Asians could produce significant negative *Z*-values (Additional file 1: Table S2). When we used CJM as the targeted, NYM as one source and combined with the above mentioned source populations, which could yield significant negative *Z*-values. When we combined northern East Asian populations (Japanese, Han, Yi, Daur, Mongolian, Hezhen, Xibo, and Northern_Han) and southern East Asians (Dai, Lahu, and She) or some Guizhou aboriginals (Shui_Duyun and Dong_Congjiang) as two ancestral sources, tests focused on LLM could produce significant negative *Z*-values (Additional file 1: Table S3). Interestingly, we did not find significant negative *Z*-values when we used NYM as the target group (Additional file 1: Table S4). The above results suggested that NYM might be the purest representative of the ancestral component of the HM populations, which was consistent with the ADMIXTURE results. We also found consistent results in the pairwise Fst (Fig. 3a).

We used pairwise IBD to measure shared haplotype counts, including worldwide representative populations (Fig. 3b), and we observed that the Miao people shared more IBD chunks with geographically close East Asians. We further performed out-group *f*_3_(studied Miao, East Asian/Southeast Asian; Mbuti) (Fig. 3c) and found that the Miaos shared more genetic drift with Atayal_Taiwan and Paiwan_Taiwan, followed by Miao from HGDP, and then the TK-speaking Dai and ST-speaking Han, Tujia, and Lahu.

To confirm the genetic patterns in PCA and ADMIXTURE and discover potential ancestral origins for studied Miao people, we conducted* f*_4_-statistics to explore the genetic affinities between the Miaos and modern East Asian populations in the form of *f*_4_(Han_Northern/Han_Southern, studied Miao; East Asian/Southeast Asian, Mbuti). The studied Miaos showed more allele sharing with geographically close populations compared to the Han_Northern (Additional file 1: Tables S5–S6). Furthermore, the studied Miao people also shared more alleles with geographically close TK, ST, AN-speaking populations, and Southeast Asian populations compared to Han_Southern. The results indicated that our studied Miao populations shared more alleles with geographically close people than with Han Chinese and these studied populations were less influenced by north-to-south gene flow and more influenced by gene flow from TK and ST-speaking populations. We also found that the studied Miao populations were influenced by Han-associated gene flow in descending order of DYM, CJM, LLM, and NYM.

We ran TreeMix to study the population split and mixing patterns between selected East and Southeast Asian populations (Fig. 3d). We found that our studied Miaos clustered with HM populations from HGDP and located between the northern Chinese populations represented by Hezhen, Daur, Xibo, and Tu ethnic groups and southern Chinese and Southeast Asian populations represented by Dai, Lahu, Paiwan_Taiwan, Atayal_Taiwan, Cebuano_Philippines, and Agta_Philippines. Specifically, our studied Miaos first gathered together and then mixed with the Altaic-speaking populations from northern East Asia and the ST-speaking populations from southern East Asia. NYM was less influenced by the indigenous people of the surrounding area than the other three Miao populations. We observed gene flow from East Asia to Agta_Philippines, from Yi to Lahu, and from ST-speaking populations to Miao, indicating that Miao from HGDP was influenced by ST-speaking populations. At the same time, the four Miao groups were less affected by ST-speaking populations, which was consistent with the distribution patterns observed in PCA, ADMIXTURE, and *f*_4_-statistics. It was also compatible with a mixed north–south pattern of HM populations and migration of HM populations to Southeast Asia in response to historical events.

### Genetic substructure among HM-speaking populations

We performed *f*_4_-statistics of the form *f*_4_(study1, study2; reference, Mbuti) to analyze whether genetic substructure existed among the four Miao populations (Additional file 1: Table S7). We found that no significant *Z*-values (*Z* > 3 or *Z* < −3) were observed in any pairs of *f*_4_(CJM, DYM/LLM/NYM; Eurasians, Mbuti), suggesting that the HM populations were genetically homogeneous relative to the northern East Asian, Southeast Asian, and southeast coastal Chinese populations. We observed significant negative *Z*-values in the *f*_4_(CJM, LLM/NYM; Chuanqing_Nayong, Mbuti), indicating that LLM/NYM shared more alleles with Chuanqing_Nayong compared to CJM. And we observed that previously reported Yi and Miao shared more alleles with LLM compared with CJM, as the observed negative values in *f*_4_(CJM, LLM; Yi/Miao, Mbuti). We also found that Dai shared more alleles with CJM compared to NYM, with significant positive *Z*-values for *f*_4_(CJM, NYM; Dai, Mbuti). These results suggested the existence of a genetic substructure among the four studied HM populations.

**Fig. 4:**
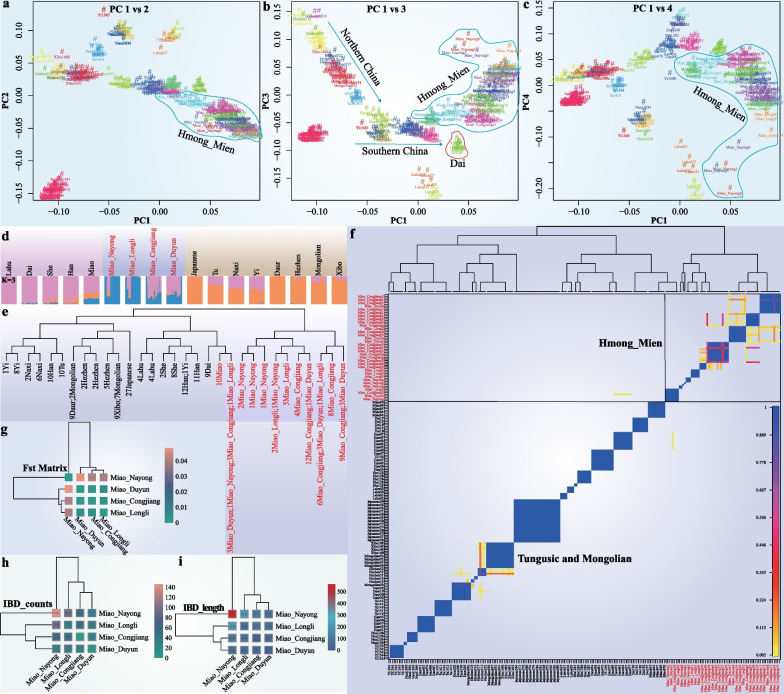
Fine-scale population genetic structure in East Asian populations. **a**–**c** PCA results based on the co-ancestry matrix revealed the genetic relationship between our studied Miao and other modern East Asians. The color showed the re-classification of the homogenous population label. **d** ADMIXTURE analysis revealed a genetic relationship between our studied Miao and other modern East Asians. **e** and **f** Clustering patterns of East Asians based on population dendrogram and the pairwise coincidence matrixes. **g** Shared genetic drift based on the pairwise Fst showed the genetic relationship between our studied HM people. **h** and **i** Number and length of shared alleles based on IBD between our studied HM people

We further inferred the fine-scale genetic structure of studied HM populations using fineSTRUCTURE (Fig. 4). The PCA based on the sharing co-ancestry matrix and ADMIXTURE results based on allele frequency spectrum indicated that our studied Miao populations formed a separate genetic branch, which was closely related to the HM populations from HGDP, Dai, and southern Han Chinese. It was distinguished from the Altaic- and ST-speaking populations in northern China and ST-speaking populations in south China (Fig. 4a–d). We further confirmed these genetic distribution patterns in a clustering model based on shared IBD fragments at the population and individual levels (Fig. 4e–f). Similar to the ADMIXTURE results, NYM and LLM were genetically closely related to each other (Fig. 4g–i).

### qpAdm-based admixture models and admixture times

The intense genetic relationship between the historic populations from southern China and the studied HM people has been demonstrated in the descriptive analyses and the quantitative *f*-statistics. We further performed two-way qpAdm models to explore the ancestral composition of our studied Miao people, with southern East Asians as the southern surrogates and ancient northern East Asians as the northern surrogates. We tested two-way admixture models and found that ancestry proportion from the ancient southern populations ranged from 0.864 ± 0.077 in the AR14K-Yiyang model to 0.372 ± 0.036 in the China_YR_LN-Cebuano_Philippines model (Additional file 1: Table S8). To further discuss whether distinct genetic contributions derived from the island and inland southern East Asians and Southeast Asians contributed to the studied populations, we then performed three-way admixture models with one source from southern East Asia (inland and island), one from northern East Asia, and another from Southeast Asia. The inferred northern sources included Neolithic middle and upper Yellow River farmers of the China_YR_MN, China_YR_LN, China_YR_LBIA, Upper_YR_LN, Shimao_LN, AR_EN, and AR19K, AR14K, AR13_10K; the southern East Asian inland sources were made up of Lahu, Dushan, Baojianshan, Longlin, Shenxian, Layi, Yiyang, BaBanQinCen and island sources of Paiwan_Taiwan, Atayal_Taiwan, Taiwan_Hanben_IA; Southeast Asian ancestral sources comprised of Thailand_IA, Laos_BA, Vietnam_N, Laos_LN_BA, Vietnam_BA. The fitted admixture models revealed that studied populations derived their primary ancestry from southern island sources and modern southern East Asian, a second from the northern sources, and the final one from the ancient southern and Southeast Asian sources (Additional file 1: Table S9). The admixture times with different ancestral populations based on MALDER suggested that north–south population interaction and admixture occurred at least in the Neolithic period and large-scale population admixture occurred between 1100 and 1600 years ago. The admixture of southern populations began at least 3500 years ago, with large-scale population admixture occurring between 1100 and 2800 years ago (Additional file 1: Table S10).

### Complex population events contributed to the patterns of genetic diversity of HM-speaking populations

**Fig. 5:**
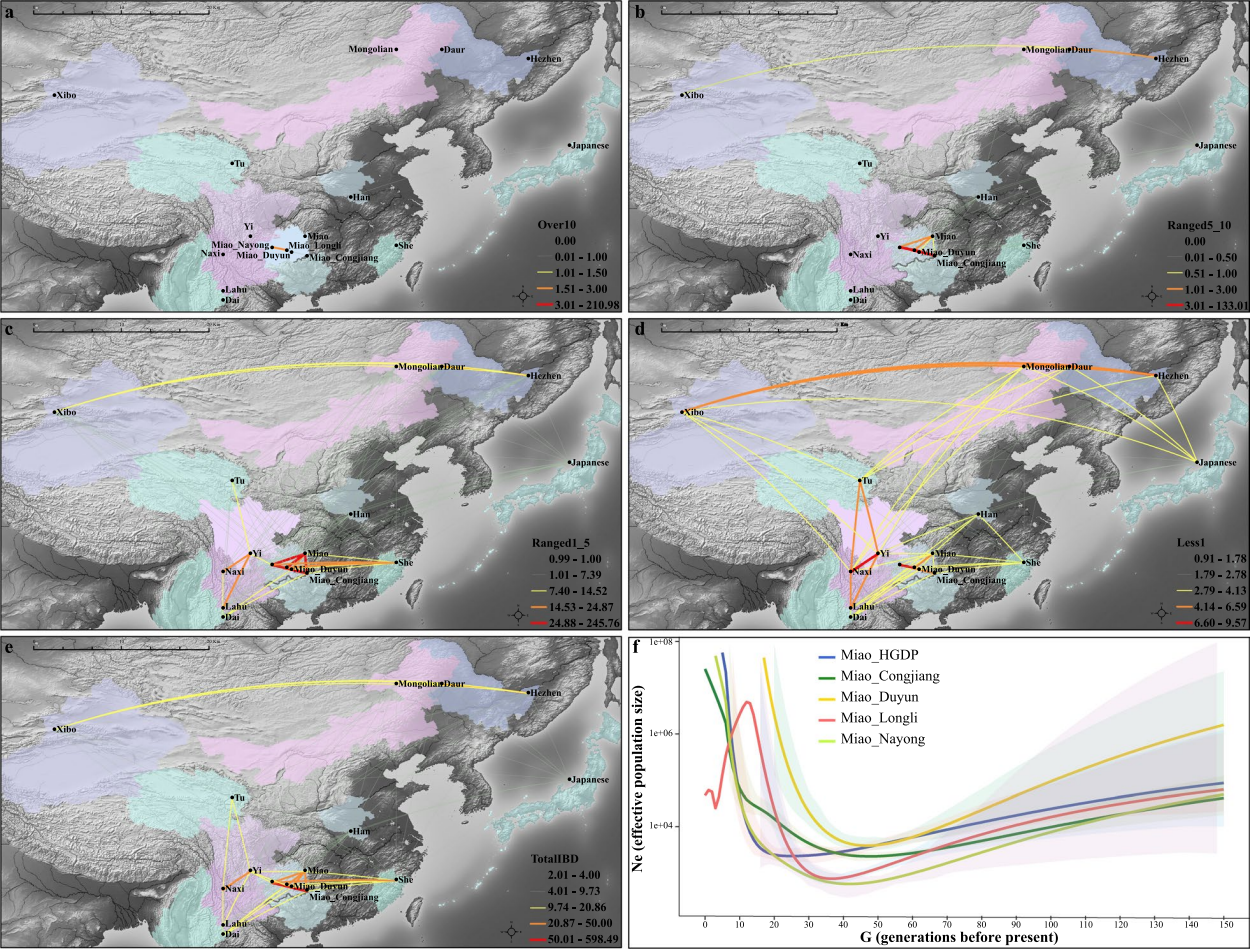
Complex population events contributed to the patterns of genetic diversity of HM groups. **a**–**e** Shared IBD fragments in different length ranges of Miaos and other populations in East Asia. **f** The effective population size of Miaos from 150 generations before the present, the Miao_HGDP has increased exponentially at about 20 generations, and our studied Miao has increased exponentially at about 40 generations

Population admixture models and descriptive analysis have shown that the Miao people were an admixture population with both northern and southern East Asian sources. Like other East Asians, admixture served as the major force to drive the formation of the gene pool of HM people. We further explored whether other additional forces contributed to the observed patterns of genetic diversity. We calculated the Runs of Homozygosity (ROH) and found that Miao people had the most significant ROH values compared with other populations. The estimated IBD fragments in different length ranges showed that Miao people had high genetic communication with each other in the past two thousand years as the more shared IBD in different categories (Fig. 5a–e). The estimated effective population size showed a plausible population bottleneck in the ancient Miao people, consistent with the observed patterns of ROH (Fig. 5f). Finally, we conducted an IBD-based admixture time estimation via fastGLOBETROTTER. We only found one population that showed the identified admixture signatures with gene flow from Han Chinese, and the other three populations showed unclear signal. The identified signatures showed that both complex admixture and population bottleneck contributed to the formation of the genetic diversity of modern HM people.

### Natural selection signatures and biological adaptation

**Fig. 6:**
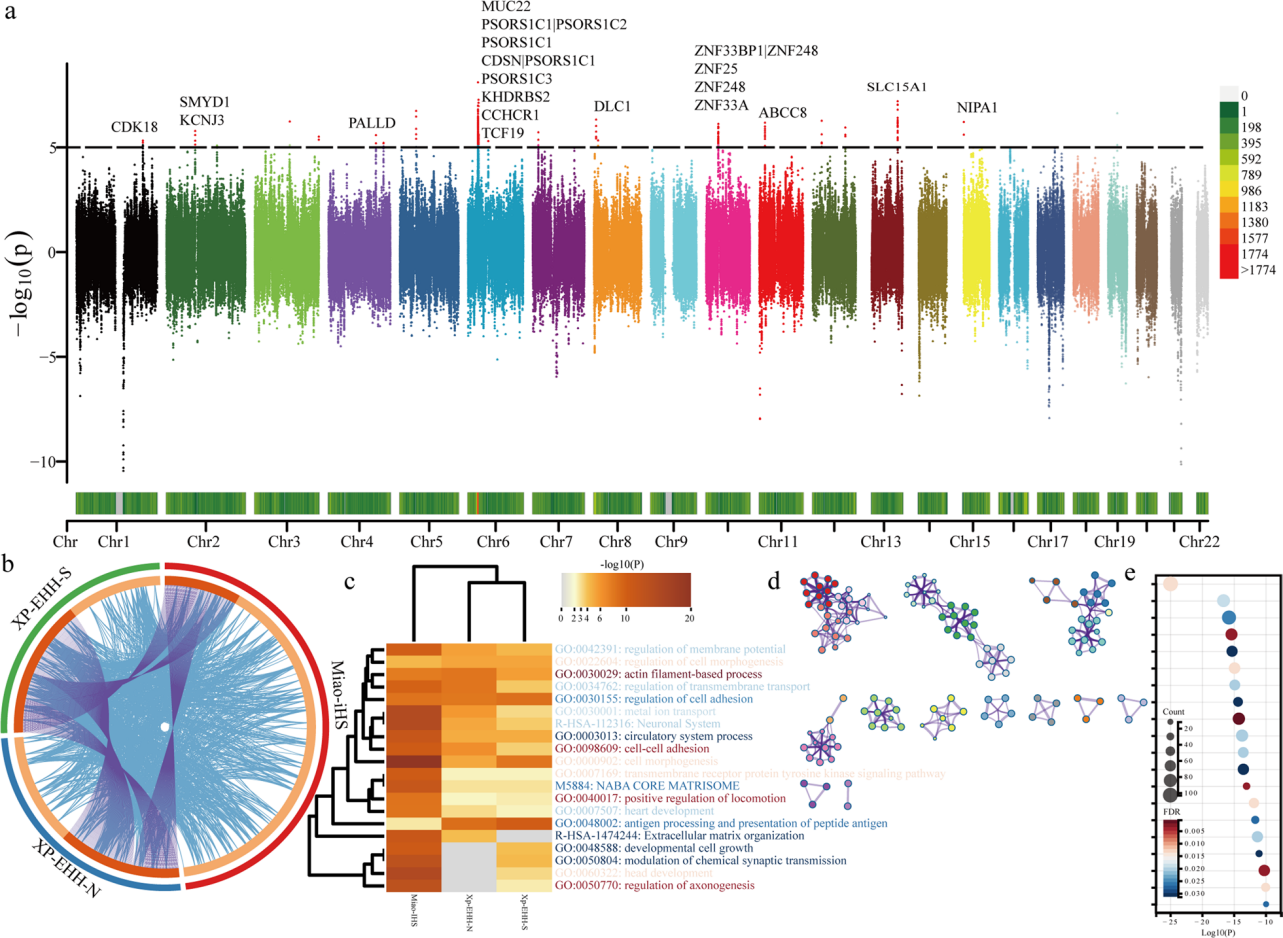
Manhattan plots showed the natural selection signatures and the results of enrichment analysis. **a** XPEHH value in the Miao populations with Han_Northern as the reference population. **b** Overlapped pattern among three selected gene lists, where purple lines link the same gene that is shared by multiple gene lists, and blue lines link genes that belong to under the same ontology term. On the inside, each arc represents a gene list, where each gene member of that list is assigned a spot on the arc. The dark orange color represents the genes shared by multiple lists, and the light orange color represents genes unique to that gene list. **c** Heatmap and dendrogram of the three gene lists for the top 20 enriched terms, colored by *P* value. **d** Enriched terms network colored by cluster-ID. **e** The enriched terms for the top 20 clusters and their representation

Genetic evidence has identified many biologically adaptive genes or pathways between ethnolinguistically diverse populations. With the increase in initial allele frequencies, adaptive mutations could confer higher immunity in target populations relative to other unexposed populations. More pathogen exposure will inevitably result in more frequent adaptive gene mutations. We screened candidate genes subjected to positive selection using integrated haplotype score (iHS) and cross-population extended haplotype homozygosity (XPEHH). We first computed XPEHH values for the Miaos with the Northern Han Chinese as a reference population and identified selection signatures on chromosomes 1, 2, 4, 6, 8, 10, 11, 13, and 15 (Fig. 6a). We observed that chromosome 1 exhibited a positive selection signal for the *CDK18* gene, which maintains genomic stability [33]. And we found positive selection on chromosome 2 for the *SMYD1* gene associated with hypertrophic cardiomyopathy stability [34] and the potassium channel arrhythmia-associated gene *KCNJ3*. We also screened for its overexpression on chromosome 4 related to the cancer gene *PALLD* [35]. We further observed strong selection signals on chromosome 6, including *MUC22*, *PSORS1C1*, *PSORS1C3*, *PSORS1C1|PSORS1C2*, *CDSN|PSORS1C1*, *CCHCR1*, *TCF19*, *KHDRBS2*. Among them, *PSORS1C1*, *PSORS1C3*, *PSORS1C1|PSORS1C2*, *CDSN|PSORS1C1*, *and CCHCR1* are genes involved in autoimmune diseases and are expressed in psoriasis, rheumatoid arthritis ankylosing spondylitis [36–38] and *PSORS1C3* is also linked to major depressive disorder suicide [38]. *MUC22* is upregulated or downregulated in lung adenocarcinoma and squamous cell carcinoma [39] and is also seen in childhood asthma Expression [40]. *TCF19* may act as a susceptibility gene for non-small cell carcinoma in the Chinese populations, interacting with tumor suppressor protein (*P53*) and playing a role in various metabolic pathways, including cancer and diabetes [41]. *KHDRBS2* has a regulatory role in microenvironmental permeability, hereditary retinopathy, and cancer [42, 43]. We also identified a positive selection signal for a tumor suppressor-associated gene (*DLC1*) on chromosome 8 [44]. We further identified positive selection signals for zinc finger protein family members (*ZNF25*, *ZNF248*, *ZNF33BP|ZNF248*, *ZNF33A*) on chromosome 10 [45]. And we identified a positive selection signal for the ATP-binding cassette transporter subfamily C member 8 (*ABCC8*) gene on chromosome 11, a variant that causes hereditary diabetes and hyperinsulinemia [46]. We also observed *SLC15A1*, a peptide transporter protein associated with digestion and absorption, on chromosome 13 [47] and a positive selection signal for the *NIPA1* gene, which causes neurodegenerative diseases in chromosome 15 [48].

We further counted XPEHH values with the southern Han Chinese as the reference group and iHS values of four Miao populations to exploit all potential candidate genes and adaptive pathways (Fig. 6b–e). We screened the top 1% of XPEHH values and normalized iHS values and enriched these genes using Metascape online software. More overlapping motifs were observed from the XPEHH screen and less from the iHS screen in the three candidate gene lists, and we could observe more functional overlap in the three candidate gene lists. Heatmap based on enrichment pathway *p* values showed genes associated with regulation of transmembrane transport (GO:0034762), actin filament-based process (GO:0030029), regulation of membrane potential (GO:0042391), regulation of cell morphogenesis (GO:0022604), regulation of cell adhesion (GO:0030155), Neuronal System (R-HSA-112316), metal ion transport (GO:0030001), cell–cell adhesion (GO:0098609), circulatory system process (GO:0003013), cell morphogenesis (GO:0000902), heart development (GO:0007507), positive regulation of locomotion (GO:0040017), NABA CORE MATRISOME (M5884), transmembrane receptor protein tyrosine kinase signaling pathway (GO:0007169), antigen processing and presentation of peptide antigen (GO:0048002), and iHS shows a strong positive selection signal for extracellular matrix organization (R-HSA-1474244), developmental cell growth (GO:0048588), modulation of chemical synaptic transmission (GO:0050804), head development (GO:0060322), regulation of axonogenesis (GO:0050770).

## Discussion

It is well known that East Asian populations account for one-fifth of the worldwide populations and have high genetic diversity. Although studies on the genetic structure and population admixture history of East Asians have increased significantly in the last two decades, the genetic diversity of East Asian populations has been studied with fewer representative populations compared to European populations. Human genomic studies have promoted a better understanding of the benefit of personal genomics and precision medicine [49, 50]. Under-representation and European bias in human genetic studies have hindered the healthy equality of ethnolinguistically diverse populations [51]. Thus, large-scale population genomic studies in under-studied regions are important and emergency [51]. As early as 2010, scholars have illuminated the patterns of the diversity of ethnicity, language, culture, and colonial history, and they also discovered the complex migration events between Southeast Asia and North Asia and the genetic relationships and various social events among East Asian hunters based on multiple evidence [1]. For a long time, scholars had studied the genetic diversity of East Asian populations through low-density genetic markers (STRs, SNPs, and Indels) and high-density genetic markers, including genome-wide SNPs and whole-genome sequencing, which have provided initial explorations of the population structure and mixing history of East Asian populations [5, 52–56]. Genetic relationships among various East Asian ethnic groups have been studied mainly using STRs, Indels, and Y-chromosomal markers, which have identified the north–south stratification and north–south genetic mixing patterns in China [57, 58]. Previous genetic studies also suggested that the distribution of genetic diversity of different ethnic groups in China was closely related to their linguistic and geographical divisions [59, 60], such as the identified genetic differentiation between highlanders and lowlanders [61]. Liu et al. genotyped forensic-related genetic markers of Tibetans on the Tibetan Plateau and combined low-density/high-density genome-wide data of modern and ancient high-altitude East Asians, and they demonstrated the close relationship between the Tibetan Plateau and Yellow River Basin populations [61].

To comprehensively characterize the genetic diversity of East Asians and develop a next-step sampling plan in the genomic cohorts in the 10K_CPGDP (10,000 Chinese Person Genomic Diversity Project) and GSRD100K^WCH^ (Genome sequencing of Rare Diseases of 100,000 people in China), we are conducting comprehensive genetic screening for the basic background of ethnolinguistically diverse populations. Here, we collected 67 Miao samples from four regions in Guizhou. Our analysis revealed genetic characteristics unique to the HM populations in Guizhou, including the genetic affinity and genetic structure of the studied HM populations with ancient and modern East Asian people as determined by PCA, East Asian-scale ADMIXTURE, and Fst. It showed that our studied HM populations were influenced by north–south gene flow. The tight clustering pattern of HM-speaking populations and the relationship between HM and Gaohuahua people were confirmed by *f*_4_-statistics, out-group *f*_3_-statistics, shared IBD fragments, and fineSTRUCTURE, and the genetic substructure among studied HM populations was determined by *f*_4_-statistics and TreeMix phylogeny.

We defined candidate genes associated with several different biological functions (locomotion, signaling, localization, regulation of biological process, and developmental process) in the HM populations (*CDK18*, *SMYD1*, *KCNJ3*, *PALLD*, *MUC22*, *PSORS1C1|PSORS1C2*, *PSORS1C1*, *CCHCR1*, *TCF19*, *PSORS1C3*, *KHDRBS2*, *CDSN|PSORS1C1*, *DLC1*, *ZNF25*, *ZNF248*, *ZNF33BP1|ZNF248*, *ZNF33A*, and *ABCC* (*ZNF33A*, *ABCC8*, *SLC15A1*, *NIPA1*)). More Denisovan ancient adaptive infiltration signals associated with immune function [62] have been confirmed in Island Southeast Asian and Oceanian populations. Interestingly, we observed natural selection signals associated with immune function in the *AGER* gene on chromosome 6 (iHS score: 3.8638 in rs2071288, 3.7444 in rs2070600), and the *RHOH* gene on chromosome 4 (3.833187 in rs73808637). Rachel et al. showed that *OCA2* and *BNC2* are genes that affect the skin pigmentation phenotype [63], but no corresponding natural selection signal was observed in the Guizhou HM populations. And we did not observe a natural selection signal for the lactase (*LCT*) [64] gene in the Guizhou HM-speaking populations. *ALDH2* and *ADH1B* are strongly associated with alcohol metabolism [65], and we only observed that the *ADH1B* gene showed a strong selectivity, reaching a maximum XPEHH score at position rs1229984 on chromosome 4 for 2.7829. The genomic selection processes observed in HM-speaking populations are related to the unique demographic history of Southwest China and the specific living environment of the Yungui Plateau. Therefore, comprehensive sampling and sequencing of the various ethnic groups in Southwest China will help us gain new insights into the genetic landscape of Southwest China and the history of migratory and adaptive changes in HM populations.

Interestingly, we found that genes closely associated with coronary heart disease (*CETP*, *LDLR*, *APOE*, and *ABCA1*) were selected in HM populations. Cholesteryl ester transfer protein (*CETP*) (iHS score of rs12597002:1.6131, and rs291044: 1.6484) is a liver-synthesized glycoprotein, and some studies have shown that *CETP* inhibitors can be used for the prevention and treatment of coronary heart disease and atherosclerosis [66]. Low-density lipoprotein receptor (*LDLR*) was selected when using the population from southern East Asia as a reference (rs2738456\rs2738458\rs2569538\rs2569537\rs14158\rs143309, XPEHH score > 2); *LDLR* was selected in the HM populations when using the northern East Asian population as a reference (rs1433099, rs14158, rs2569537, rs5929, XPEHH score > 2), and *LDLR* achieves modulation of atherosclerosis by regulating cholesterol homeostasis (Li et al. 2021; Go and Mani 2012). ATP-binding cassette subfamily A member 1 (*ABCA1*) was selected in the HM populations when southern East Asians were used as a reference (rs2472377, rs2472508, rs10991415, rs4149310, rs4149311, rs2487052, XPEHH score > 1.5, and rs62566032, iHS score = 2.6302), and *ABCA1* prevents cardiovascular disease by promoting the efflux of intracellular cholesterol and phospholipids, controlling the rate-limiting step of reverse cholesterol transport and by inhibiting inflammation and maintaining lipid homeostasis.

## Conclusion

We provided genome-wide SNP data for HM-speaking Miaos in Guizhou Province and identified a distinct genetic branch of HM populations. Our results revealed a mixed pattern of HM people. Han-related genetic materials influenced Guizhou Miao people to varying degrees, and our studied Miaos were all influenced by a smaller stream of exotic genetic material than those in previous studies. Our results suggested the existence of genetic substructures among our studied Miaos, in which Han-related genes influenced the DYM and CJM significantly. At the same time, the LLM and NYM received more gene flow from the ancient people of Guangxi. Our results showed that NYM is more representative of the ancestry of the HM populations than Gaohuahua. We provided genetic evidence for a north–south East Asian mixing pattern in the HM populations and ALDER‐based admixture times further inferred the onset of north–south admixture in East Asians since Neolithic period and the timing of large-scale population mixing in the south occurred between 1100 and 2800 years ago. Finally, we identified hundreds of loci involved in the immune function disorders located on chromosome 6 and *ADH1B*, *CETP*, *LDLR*, and *ABCA1* associated with alcohol and coronary heart disease.

## Methods and materials

### Sample collection, genotyping, and quality control

We collected blood samples from 67 unrelated individuals from four counties of Guizhou Province in southwestern China (CJM, NYM, DYM, and LLM, Fig. 1a). These samples were randomly selected, and written informed consent was provided before participating in this study. Their parents and grandparents were indigenous residents for at least three generations, and they had no consanguineous marriages. This study was reviewed and approved by the Medical Ethics Committee of Guizhou Medical University and North Sichuan Medical College. Besides, the procedure followed the recommendations of the Helsinki Declaration as revised in 2000 [67]. We genotyped these samples using the Affymetrix array containing approximately 699,896 SNPs. Quality control was performed by Plink 1.9 [68] with the option “-missing 0.05 -HWE 0.0001” to calculate the SNP call rate. SNPs that failed to reach the missing threshold of 0.05 and an alpha level of 0.0001 for HWE testing were removed.

### Relatedness analysis

Before merging data, we initially performed KING2 [34] to check individual relationships by calculating kinship coefficients [32]. All unrelated samples were kept for the following analyses.

### Data merging

We merged our newly generated data of 67 samples with the previously published Human HO and the 1240K datasets from the Allen Ancient DNA Resource (AADR) via the mergeit tool in the EIGENSOFT [69] with the strandcheck parameter setting of Yes to remove the AT/CG SNPs (https://reich.hms.harvard.edu/datasets). We generated three combined datasets that was used in subsequent analysis. The merged HO-based dataset with more present-day reference populations covered 95,342 SNPs variants, and the merged 1240K-based dataset covered 245,627 SNPs variants. We also merged other recently published ancient East Asian people in our analysis [8, 70, 71]. To obtain one high-density dataset, we combined our data with whole-genome sequencing data from Oceania, Asia, Europe, and Africa to form a standard merged WGS) dataset [62, 72] and used it to explore complex admixture models. The combined WGS dataset covered 679,602 SNPs. We used low-density SNP data in Fst, PCA and ADMIXTURE analyses and the high-density SNP data in *f*_3_-statistics and *f*_4_-statistics.

### Principal component analysis

We performed three levels of PCA analyses, focusing on the relationship between East and Southeast Asian populations and the HM populations. We used the smartpca program of the EIGENSOFT package [69] with default parameters of numoutlieriter: 0 and lsqproject: YES to conduct PCA analysis and projected ancient samples onto the PCA axes generated by present-day samples. The PCA at the East and Southeast Asian level includes 46 individuals from 5 Tungusic and Mongolic-speaking populations in northern China, 35 individuals from 4 Tibeto-Burman-speaking populations, 9 Dai individuals from TK-speaking populations, and 33 Han Chinese from South China, 27 individuals from Japan, 19 individuals from 2 AN-speaking populations, 40 Ayta and Agta individuals from Southeast Asia, and 87 individuals from 6 HM-speaking populations [62, 72]. PCA at the Chinese level includes 410 individuals from 51 populations from ancient Northwest China, Northeast China, Middle Yellow River, Southeast China coastal and island, modern Altaic, ST, and HM-speaking populations [8, 70, 71].

### Model-based ADMIXTURE analysis

We performed ADMIXTURE [73] based on the model-based maximum likelihood (ML) clustering algorithm to estimate the ancestry of individuals and determine the population structure. We trimmed SNPs of strong linkage disequilibrium with the parameters “-indep-pairwise 200 25 0.4” using Plink [68] before analyzing the admixture. We re-ran ADMIXTURE 100 times with default tenfold cross-validation (− CV = 10), with the number of ancestral populations from *K* = 2 to *K* = 10 in bootstrap sequences with different random seeds. We selected the best run based on the highest log-likelihood and used CV values to identify an “optimal” number of clusters. We then used AncestryPainter [74] to visualize the individual and population ancestry.

### Admixture ***f***_3_-statistics and out-group ***f***_3_-statistics

We used the qp3Pop (version 435) package in ADMIXTOOLS software [75] to evaluate the possible admixture signatures in Guizhou Miao via the admixture-*f*_3_-statistics in the form *f*_3_(X, Y; studied Miao). Target populations with negative *f*_3_-values and *Z*-scores less than − 3 were regarded as mixed populations of two ancestral people related to *X* and *Y*. We then evaluated the genetic affinity between studied Miao and other reference populations via the out-group *f*_3_-statistics in the form *f*_3_(Reference, studied Miao; Mbuti).

### ***f***_4_-Statistics

We employed the qpDstat program in ADMIXTOOLS to obtain *f*_4_-statistics with default parameters [75]. We calculated the *f*_4_-statistics in the form *f*_4_(Reference1, studied Miao; Reference2, Mbuti) to test whether the studied Miao shared more gene flow with the reference1 population compared with the reference2 population or shared more alleles with Reference2 compared with Reference1. A significant negative Z-score smaller than − 3 indicated that the studied Miao shared more alleles with reference2 populations compared with reference1 populations. The *f*_4_-statistics in the form *f*_4_(studied Miao1, studied Miao2; Reference, Mbuti) were used to test the genetic difference among the studied geographically different Miao.

### MALDER

To explore the admixture signatures and fit the potential admixture model with admixture times, we used MALDER to estimate the generations since the admixture events occurred [76] with the default parameters.

### qpAdm

We used ancient northern East Asians as the northern surrogate of the ancestral source and used southern East Asians as the southern surrogate of the ancestral source to model the formation of studied Miao people via qpAdm [77]. We used the following populations as out-groups: Mbuti, Iran_GanjDareh_N, Italy_North_Villabruna_HG, Mixe, Papuan, Onge, and Agta_Philippines.

### FineSTRUCTURE analysis based on the sharing haplotypes

We phased 1049 genomes from 47 populations with the default parameters using the SHAPEIT software [78] as well as computed the sharing haplotypes using the ChromoPainterv2 software [79]. Fine-scale population structures were explored using fineSTRUCTURE (version 4.0) according to the co-ancestry matrix [79].

### Haplotype-based admixture sources, proportions, and dates

We used ChromoPainter v2 [79] to estimate the copied vector, paint all targeted individuals with different ancestral surrogates, and used fastGLOBETROTTER to identify, describe, and date the admixture events with the recommended parameters [80].

### Maximum likelihood tree

We generated the ML admixture graph, with the migration events from 1 to 10, through TreeMix to further explore the relationship between the newly studied Miao and modern southern Chinese and Southeast Asian populations [81].

### IBD estimation and effective population size

We used the refined IBD [82] to estimate the pairwise IBD among individuals. We then calculated the average total IBD among populations based on the length of a single IBD less than or equal to one, ranging from one to five, five to ten, and over ten. We also used Plink to estimate the individual-level ROH) to show the status of the interbreeding coefficient [68]. IBDNe was used to estimate the effective population size among geographically diverse Miao people [83].

### Natural selection indexes of XPEHH and iHS estimation

We used the R package of REHH [84] to compute the iHS and XPEHH. Both northern Han Chinese and southern Han Chinese were used as references in the estimation of XPEHH.

## Supplementary Information

**Additional file 1. Table S1.** Z-scores of admixture-*f*_3_ in the form *f*_3_(X, Y; Miao_Longli). **Table S2**. Z-scores of admixture-*f*_3_ in the form *f*_3_(X, Y; Miao_Duyun). **Table S3**. Z-scores of admixture-*f*_3_ in the form *f*_3_(X, Y; Miao_Congjiang). **Table S4**. Z-scores of admixture-*f*_3_ in the form *f*_3_(X, Y; Miao_Nayong). **Table S5**. Results of *f4*-statistics in the form *f*_4_(Northern Han, Four Miao populations; reference populations, Mbuti). **Table S6**. Results of *f*_4_-statistics in the form *f*_4_(Han, Four Miao populations; reference populations, Mbuti). **Table S7**. Results of *f*_4_-statistics in the form *f*_4_(Miao_Congjiang, Four Miao populations; reference populations, Mbuti). **Table S8**. Two-way admixture models for the formation of Miao people. **Table S9**. Three-way admixture models for the formation of Miao people. **Table S10**. MALDER results showed the admixture times of Miao people.

## Data Availability

The genome-wide variation data were collected from the public dataset of Allen Ancient DNA Resource (AADR) (https://reich.hms.harvard.edu/allen-ancient-dna-resource-aadr-downloadable-genotypes-present-day-and-ancient-dna-data). The new-generated allele frequency data have been submitted to the public database. (The link will be available when this work is accepted.)
