# Correction: Genome-wide allele and haplotype-sharing patterns suggested one unique Hmong–Mein-related lineage and biological adaptation history in Southwest China

**DOI:** 10.1186/s40246-023-00477-5

**Published:** 2023-03-30

**Authors:** Guanglin He, Jiawen Wang, Lin Yang, Shuhan Duan, Qiuxia Sun, Youjing Li, Jun Wu, Wenxin Wu, Zheng Wang, Yan Liu, Renkuan Tang, Junbao Yang, Chao Liu, Buhong Yuan, Daoyong Wang, Jianwei Xu, Mengge Wang

**Affiliations:** 1 Institute of Rare Diseases, West China Hospital of Sichuan University, Sichuan University, Chengdu, 610041 China; 2 College of Forensic Medicine, Guizhou Medical University, Guiyang, 550004 China; 3 School of Basic Medical Sciences, North Sichuan Medical College, Nanchong, 637000 China; 4 Department of Forensic Medicine, College of Basic Medicine, Chongqing Medical University, Chongqing, 400331 China; 5 Congjiang People’s Hospital, Congjiang, 557499 China; 6 Department of Pharmacology, School of Basic Medicine, Guizhou Medical University, Guiyang, 550004 China; 7 Faculty of Forensic Medicine, Zhongshan School of Medicine, Sun Yat-Sen University, Guangzhou, 510275 China; 8 Institute of Forensic Medicine, School of Basic Medical Sciences and Forensic Medicine, Sichuan University, Chengdu, 610000 West China China; 9 Longli People’s Hospital, Longli, 551299 China; 10 Nayong Guohua Yixin Hospital, Nayong, 553306 China

**Correction: Human Genomics (2023) 17:3** 10.1186/s40246-023-00452-0

Following publication of the original article [1], the authors reported that the name of Guanglin He needed to be moved from the last to the first of the authorship.

The correct authorship has been provided in this correction.

The original article [1] has been corrected.
